# Squamous Cell Carcinoma Arising in a Zenker’s Diverticulum

**DOI:** 10.7759/cureus.53583

**Published:** 2024-02-04

**Authors:** Praveen Agarwal, Nitish Jain, Sourabh Jindal, Vivek Goel, Pradeep Jain

**Affiliations:** 1 Gastrointestinal Surgery, Fortis Hospital Shalimar Bagh, New Delhi, IND; 2 Gastroenterology and Hepatology, Fortis Hospital Shalimar Bagh, New Delhi, IND

**Keywords:** synchronous cancers, video-assisted thoracoscopic surgery (vats), metastatic lung nodule, squamous cell carcinoma (scc), zenker's diverticulum

## Abstract

Squamous cell carcinoma (SCC) developing in a Zenker's diverticulum (ZD) is an uncommon condition. The preferred treatment for SCC in the pharyngeal pouch is complete diverticulum resection. Only histopathological evaluation of the pouch can rule out SCC. Here, we present a case of a 62-year-old male patient, who was evaluated for repeated episodes of aspiration and dysphagia, and diagnosed to have a large ZD, the patient underwent Zenker’s diverticulectomy with cricopharyngeal myotomy with wide margins due to clinically suspicious specimen. Histopathological examination revealed well-differentiated SCC arising within ZD, involving the whole thickness of the wall and almost touching the serosa (1 mm). The patient developed metastatic lung nodule on PET-CT, so metastatic lung nodule was excised with video-assisted thoracoscopic surgery (VATS), and chemotherapy and immunotherapy were given. On follow-up imaging patient is tumor-free to date, two years after the surgery. The occurrence of synchronous or metachronous lung cancer makes it one of the rarest cases.

## Introduction

Abraham Ludlow first described pharyngeal pouches in 1769. Over a century later, in 1877, Zenker provided a comprehensive clinical pathological description [1]. Squamous cell carcinoma (SCC) arising in Zenker’s diverticulum (ZD) is a rare condition. The incidence ranges from 0.3 to 7% [2]. ZD is accompanied by complications like ulceration and recurrent aspiration pneumonitis, which can be treated by surgery [3]. It is crucial to perform an early assessment to rule out the development of SCC. Diverticulectomy is advised in symptomatic patients to reduce the risk of Zenker's carcinoma [4]. Here, we report a rare case of ZD with SCC in the pouch along with a metastatic lung lesion.

## Case presentation

**Figure 1:**
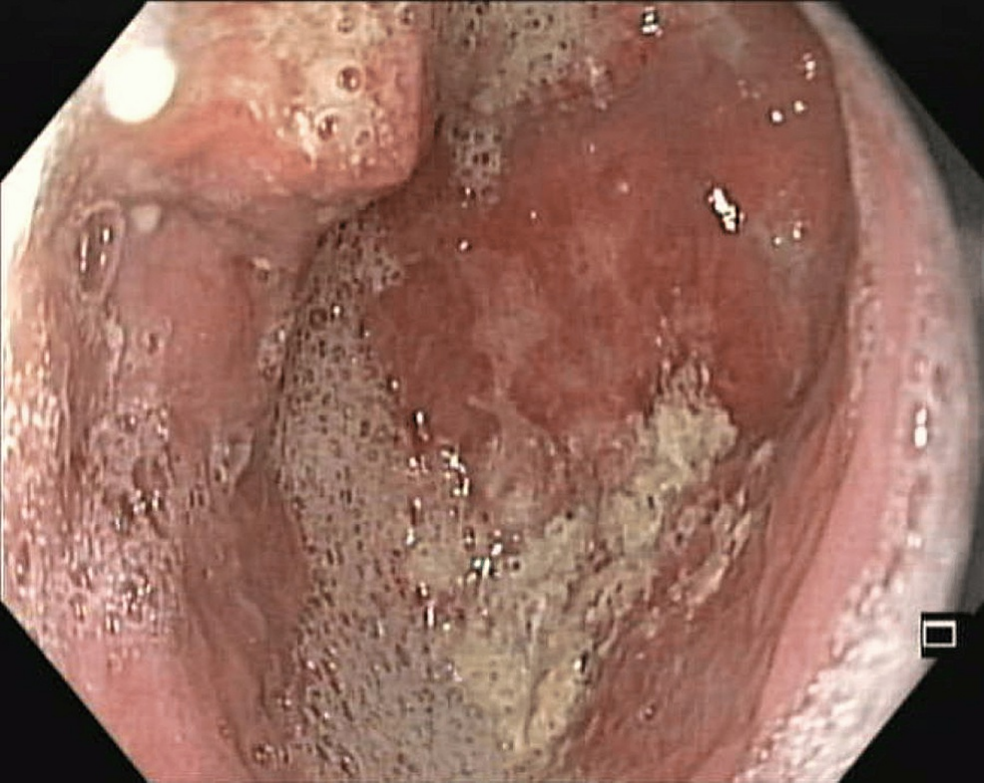
Upper gastrointestinal (GI) endoscopy showing a large ZD. ZD: Zenker's diverticulum

**Figure 2:**
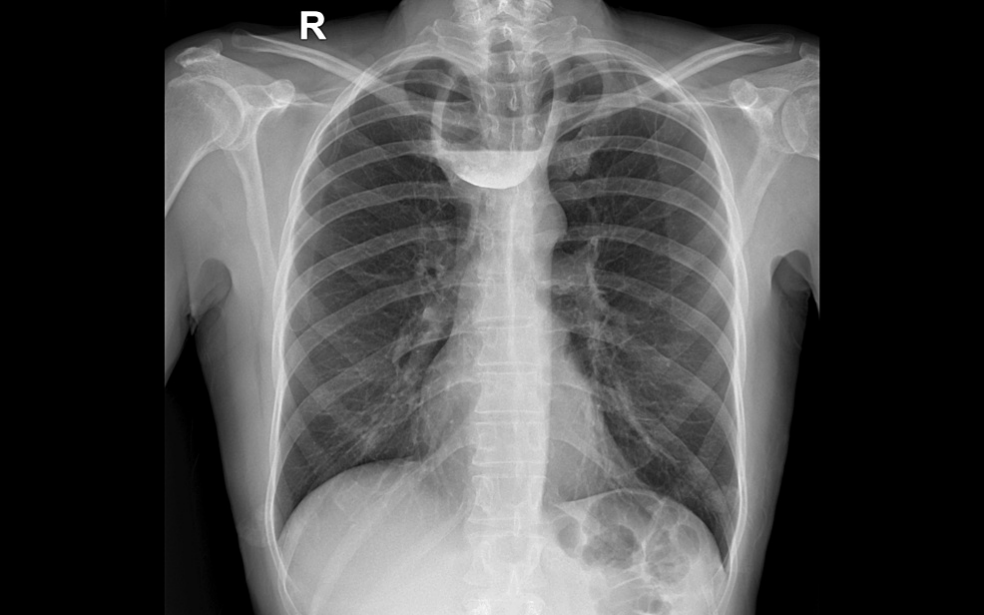
Barium swallow, anteroposterior view showing large diverticulum at the upper end of esophagus.

A 62-year-old male patient with no comorbid illness presented with a history of dysphagia and regurgitation for three years. He had repeated episodes of aspiration and worsening symptoms for the past few months. There was no history of smoking or alcohol abuse. Systemic examinations were unremarkable. All laboratory investigations were within normal limits. Upper gastrointestinal (GI) endoscopy showed a large ZD (Figure 1). Barium swallow also showed ZD (Figure 2).

**Figure 3:**
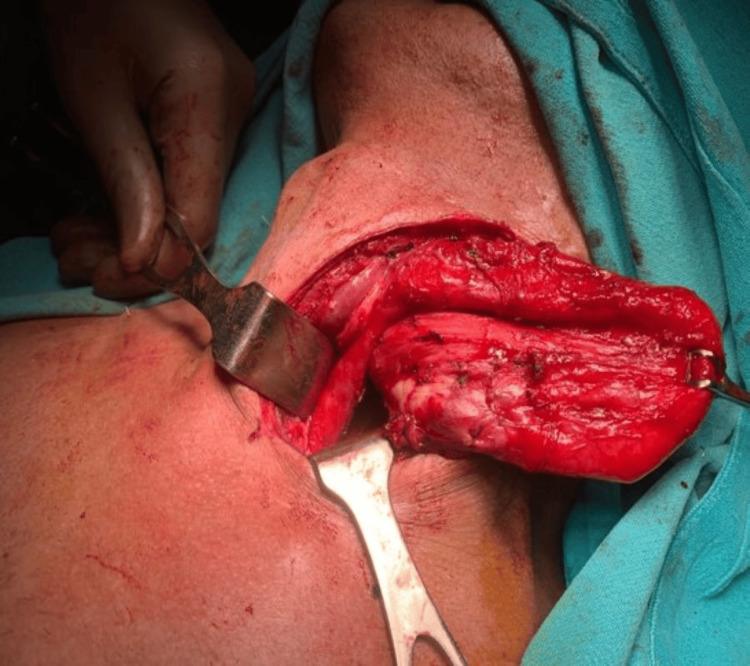
Intraoperative picture showing a large diverticulum in the upper part of esophagus.

**Figure 4:**
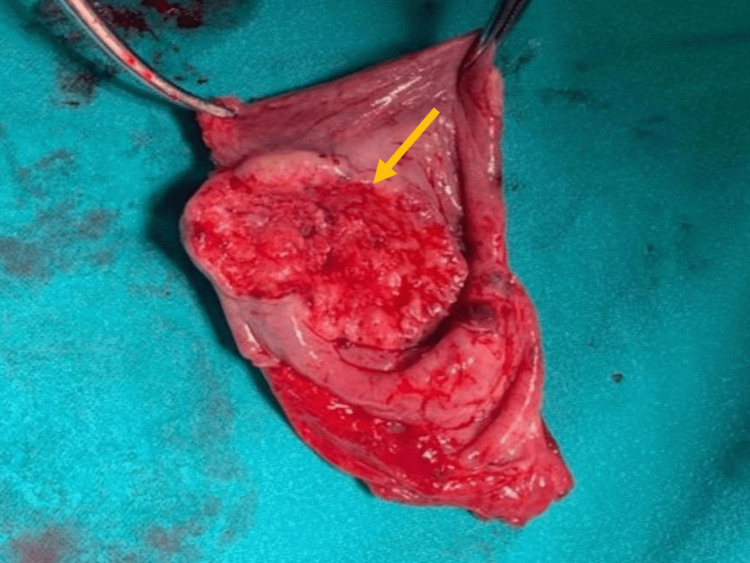
Gross image of the diverticulectomy specimen. Arrow showing ulcerated thick wall area in the wall of diverticulum.

Contrast-enhanced computed tomography (CECT) of the chest showed features suggestive of aspiration pneumonitis with a large oral contrast-filled structure with hypodense contents and air-fluid levels in the posterior mediastinum. In view of the above findings, he underwent Zenker’s diverticulectomy with a cricopharyngeal myotomy in September 2021. Intraoperatively, there was a large upper esophageal diverticulum of size 10 cm reaching the upper neck with its thickened posterior wall (Figures 3, 4).

**Figure 5:**
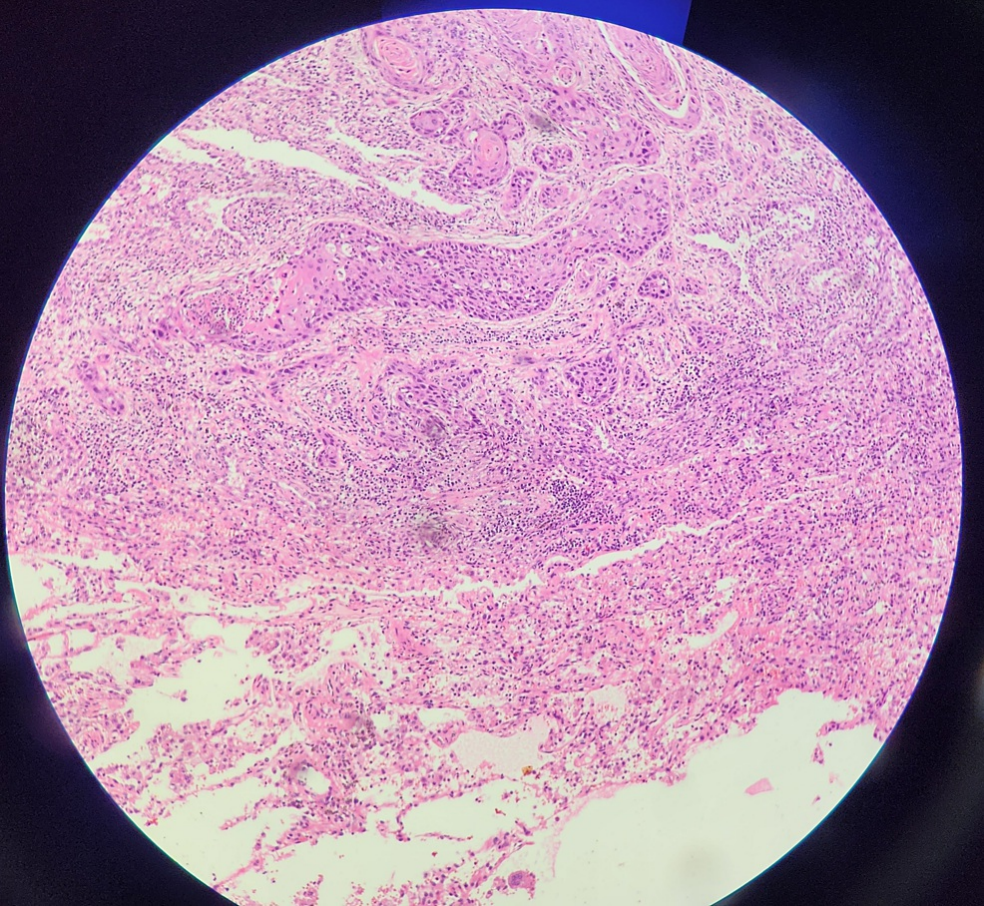
Microscopic image of the ulcerated lesion of diverticulum showing well-differentiated squamous cell carcinoma.

In view of the suspicion of malignancy due to the thick-walled diverticulum, wide margins were taken as there is no data in the literature regarding esophagectomy in SCC in ZD and the absence of patient consent for esophagectomy. The patient had an uneventful postoperative recovery except for a spike in fever on postoperative day (POD) seven. A CECT neck with oral contrast was done to rule out any leak from the repaired site, which was normal. In view of the high suspicion of leak, a barium swallow was done on POD nine, which showed a tract of contrast opacification adjacent to the upper thoracic esophagus, which was managed conservatively. On POD 12, he was discharged home on an oral soft diet. Histopathology of the resected specimen showed well-differentiated SCC arising within ZD, involving the whole thickness of the wall and almost touching the serosa (1 mm); the closest resection margin was 0.3 cm (Figure 5).

**Figure 6:**
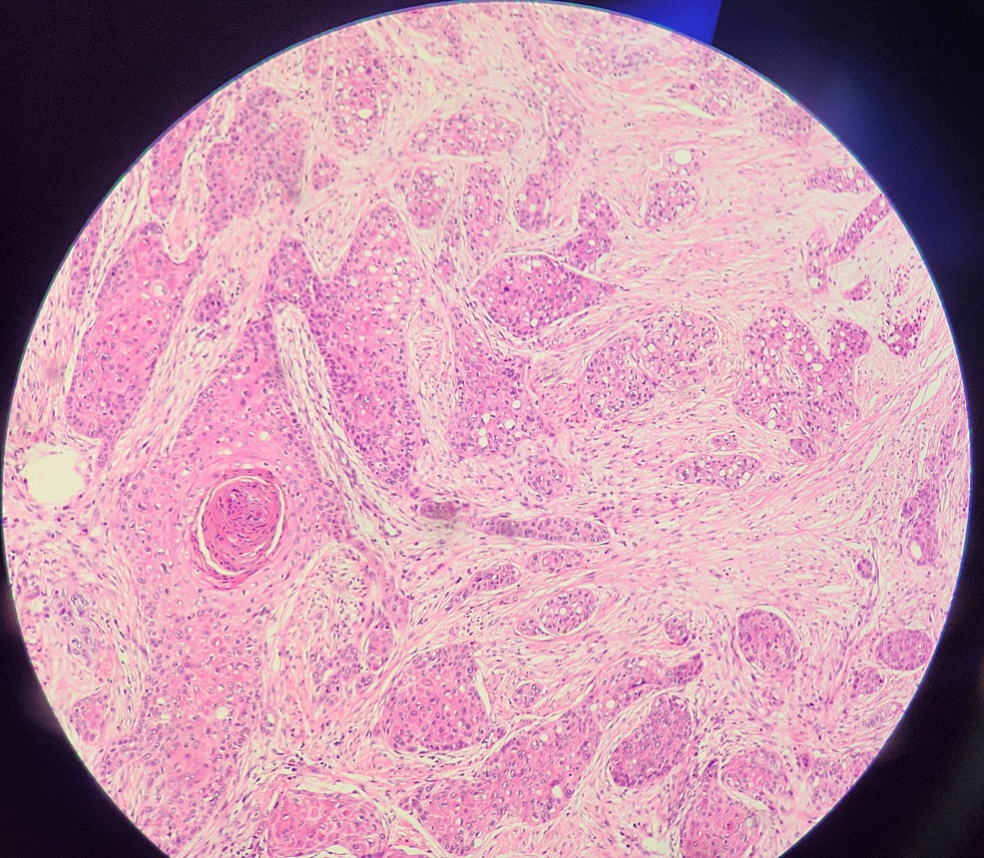
Microscopic image of lung nodule showing adenosquamous carcinoma of the lung.

As the tumor margin was closed at one site, the case was discussed on the tumor board, and he was planned for radiotherapy (RT). After six weeks of surgery, whole-body positron emission tomography (PET-CT) was done, which showed postsurgical status with fibrotic thickening in the upper thoracic esophagus at the level of D1 vertebra, with no focal abnormal hypermetabolic thickening at the postoperative surgical site, and an esophagus with hypermetabolic small area of consolidation (2 cm) in the right lung upper lobe in the posterior segment. CT-guided fine needle aspiration cytology was done on the lung lesion and it was positive for atypical cells, suggesting malignancy. Again, the case was discussed on the tumor board. In view of oligometastatic disease, the decision was taken for the resection of lung nodule. He underwent thoracoscopic resection of the right upper lobe lung nodule (wedge resection). The final biopsy of the resected lung nodule was adenosquamous carcinoma of the lung with negative surgical margins (Figure 6).

Immunohistochemical (IHC) of the lung nodule specimen was done, which confirmed adenosquamous pathology. P63 and P40 were positive in the squamous component and TTF-1, NAPSIN A, and mucicarmine were positive in the adeno component of the tumor.

Again, a diagnostic dilemma arose as to whether the lung lesion was a second primary or a metastatic lesion from the SCC of the ZD. Further next-generation sequencing was done and found to have similar mutations in both lesions; PD-L1: positive 50%, microsatellite instability (MSI): stable, and tumor mutation burden (TMB): low (5). In view of metastatic disease, the patient was started on chemotherapy with immunotherapy (pembrolizumab and nab-paclitaxel + carboplatin) and followed by a PET-CT scan. To date, he has completed 22 cycles of chemotherapy and 23 doses of pembrolizumab. After two years of surgery, the patient is disease-free, with no evidence of local and distant recurrence. To the best of my knowledge, this is a rare case of SCC with lung metastasis in ZD.

## Discussion

ZD is a pharyngoesophageal protrusion of the hypopharynx through the Killian's triangle. Advanced age and male gender are risk factors for developing ZD [5]. Its prevalence ranges from 0.01 to 0.11% [3]. ZD is associated with complications like aspiration pneumonia, ulceration and bleeding, fistula formation between diverticulum and adjacent organs, vocal cord paralysis, and SCC in very rare cases [6]. Halstead provided the first description of an SCC occurrence in a ZD in 1904 [3]. The diagnosis of SCC in the pouch is usually made after a full histopathological examination of the specimen after a complete surgical resection [7,8]. The old age of the patient, large pouch size, and long duration are risk factors for developing cancer of the pouch. There is an increased risk of cancer from prolonged and direct irritation and inflammation of the pouch due to the high frequency of food retention and manual emptying of the pouch by digital pressure [7,8].

Signs and symptoms like changes in the nature of dysphagia, rapid progression of dysphagia, pain, and weight loss suggest malignancy [8]. Most of the cases of ZD are diagnosed by contrast imaging and upper gastrointestinal (UGI) endoscopy [7]. Treatment is aimed at relieving the symptoms of the patient. The most common modality used is endoscopic stapling or diverticulectomy [3].

In our case, the histopathological examination (HPE) report showed closed tumor margins and metastatic lung nodules on PET-CT; hence, we underwent excision of the metastatic lung nodule followed by chemotherapy and immunotherapy. On follow-up imaging, the patient is tumor-free to date.

## Conclusions

In summary, SCC developing in ZD is very uncommon. High clinical suspicion is important to rule out a malignant transformation. Most cases of ZD are diagnosed by contrast imaging and UGI endoscopy, but it could miss carcinoma in situ, where histopathological assessment of the complete resected pouch is the definitive modality of diagnosis. We advise a complete surgical excision and frozen section of the pouch for all patients who have clinical suspicion of malignancy on the table, to relieve the symptoms, prevent the complications of delayed recognition, and maximize the therapeutic intervention. The occurrence of synchronous or metachronous lung cancer makes it one of the rarest cases.
